# Transcriptome Sequencing Reveal That Rno-Rsf1_0012 Participates in Levodopa-Induced Dyskinesia in Parkinson’s Disease Rats via Binding to Rno-mir-298-5p

**DOI:** 10.3390/brainsci12091206

**Published:** 2022-09-07

**Authors:** Chun-Lei Han, Qiao Wang, Chong Liu, Zhi-Bao Li, Ting-Ting Du, Yun-Peng Sui, Xin Zhang, Jian-Guo Zhang, Yi-Lei Xiao, Guo-En Cai, Fan-Gang Meng

**Affiliations:** 1 Department of Neurosurgery, Beijing Tiantan Hospital, Capital Medical University, Beijing 100070, China; 2 Beijing Key Laboratory of Neurostimulation, Beijing 100070, China; 3 Department of Functional Neurosurgery, Beijing Neurosurgical Institute, Capital Medical University, Beijing 100070, China; 4 Department of Neurosurgery, Liaocheng People’s Hospital, Liaocheng 252004, China; 5 Department of Neurology, Fujian Medical University Union Hospital, Fuzhou 350001, China; 6 Fujian Key Laboratory of Molecular Neurology, Institute of Clinical Neurology, Institute of Neuroscience, Fujian Medical University, Fuzhou 350122, China; 7 Chinese Institute for Brain Research, Beijing 102206, China

**Keywords:** Parkinson’s disease, levodopa-induced dyskinesia, circular RNA, next-generation sequencing

## Abstract

Levodopa-induced dyskinesia (LID) is a common complication of chronic dopamine replacement therapy in the treatment of Parkinson’s disease (PD), and a noble cause of disability in advanced PD patients. Circular RNA (circRNA) is a novel type of non-coding RNA with a covalently closed-loop structure, which can regulate gene expression and participate in many biological processes. However, the biological roles of circRNAs in LID are not completely known. In the present study, we established typical LID rat models by unilateral lesions of the medial forebrain bundle and repeated levodopa therapy. High-throughput next-generation sequencing was used to screen circRNAs differentially expressed in the brain of LID and non-LID (NLID) rats, and key circRNAs were selected according to bioinformatics analyses. Regarding fold change ≥2 and *p* < 0.05 as the cutoff value, there were a total of 99 differential circRNAs, including 39 up-regulated and 60 down-regulated circRNAs between the NLID and LID groups. The expression of rno-Rsf1_0012 was significantly increased in the striatum of LID rats and competitively bound rno-mir-298-5p. The high expression of target genes PCP and TBP in LID rats also supports the conclusion that rno-Rsf1_0012 may be related to the occurrence of LID.

## 1. Introduction

Parkinson’s disease (PD) is a chronic, progressive disease mainly affecting middle-aged and elderly people. It is characterized by tremors, rigidity, decreased movement, abnormal postural reflex, and autonomic nervous dysfunctions. It is the second most common neurodegenerative disease in the world after Alzheimer’s disease and affects approximately 1% of adults over age 60 [1]. The main pathological changes of PD include the loss of dopaminergic neurons and the formation of Lewy bodies in the substantia nigra of the midbrain. The pathogenesis of PD is unclear, but mitochondrial dysfunction, oxidative stress, altered protein handling, and inflammation may contribute to nigral dopaminergic cell death [2].

At present, levodopa is still the first choice for PD treatment. However, with progression of the disease, long-term levodopa treatment may show decreased efficacy and cause symptom fluctuations and motor complications. Studies have suggested that 8–45% of PD patients experienced dyskinesia after 4–6 years of treatment [3,4,5] with a disability as high as 43% [6]. It is believed that the occurrence and development of LID are related to the inherent lesions of the nigrostriatal regions and the pulsatile delivery of levodopa, involving a variety of neural signaling pathways and changes in brain network electrophysiological activities [7]. At present, the exact mechanism of LID is not clear, and effective treatment strategies are also lacking in clinical practice.

Circular RNA is a newly discovered non-coding RNA with a closed circular structure, which is resistant to exonuclease and abundant in the whole transcriptome. The functions of most circRNAs remain unexplored, but many circRNAs exert important biological functions by acting as microRNAs or protein inhibitors, to regulate protein functions or to be translated [8]. CircRNAs are evolutionarily conserved and are more stable than linear RNAs, so circRNAs have enormous potential to be diagnostic and prognostic biomarkers [9]. Studies have shown that circRNAs are closely related to brain development [10], nervous system tumors [11], Alzheimer’s disease [12], and PD [13]. For example, Hanan et al. found that circSLC8A1, which carries seven binding sites for miR-128 and is strongly bound to the microRNA effector protein, Ago2, may affect oxidative stress in PD by regulating miR-128 [13].

There is growing evidence suggesting the potential role of circRNAs in nervous system disease. However, there are no reports on the role of circRNAs in LID. Here we studied the circRNA expression profile of the striatum of LID rats by high-throughput sequencing to identify circRNAs related to LID for further study.

## 2. Materials and Methods

### 2.1. Animals

Male specific pathogen-free (SPF) Sprague–Dawley (SD) rats (250−300 g) were obtained from Vital-River Experimental Animal Technology (Beijing, China). Animals were maintained in a temperature-controlled room on a 12/12 h light/dark cycle with ad libitum access to standard food and water. Animal experiments were conducted according to the Chinese Animal Welfare Act and Guidance for Animal Experimentation of Capital Medical University. The study protocol was approved by the Ethics Committee of Beijing Neurosurgical Institute, Capital Medical University (Protocol No.: AEEI-2018-200).

### 2.2. 6-OHDA Lesion and L-DOPA Administration

Rats were anesthetized with 2−3% isoflurane through an animal anesthesia ventilator system (RWD Life Science, Shenzhen, China) and placed in a stereotaxic frame (David Kopf Instruments, Tujunga, CA, USA). Based on previous PD rat model studies, rats were unilaterally lesioned by injection of 6-OHDA (12 μg/2.4 μL in 0.02% ascorbate in saline (162957, Sigma-Aldrich, St. Louis, MO, USA)) into the medial forebrain bundle (MFB) (from bregma: anterior posterior (AP): −3.6 mm, medial lateral (ML): −1.8 mm; dorsal ventral (DV): −8.2 mm from the skull)) using a Hamilton syringe (88000, Hamilton, Reno, NV, USA). The 6-OHDA was injected at a rate of 1 μL/min, and the needle was left in place for an additional 5 min to allow diffusion of 6-OHDA before being slowly retracted. To determine lesion efficacy, turning behavior was recorded 3 weeks later over a 90 min period after injection of apomorphine (0.5 mg/kg by subcutaneous injection (16094, Cayman, Ann Arbor, MI, USA)) [14]. The control rats received a sham lesion using saline.

**Figure 1:**
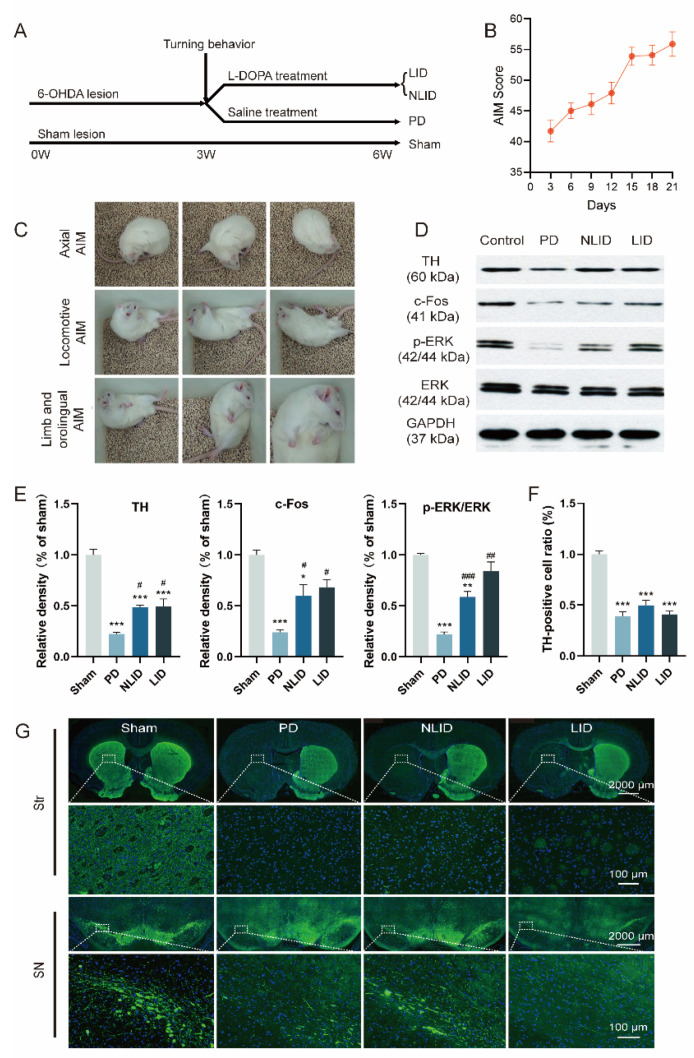
Validation of the levodopa-induced dyskinesia (LID) rat model. (**A**) Experimental timeline. A unilateral Parkinson’s disease (PD) rat model was established by 6-OHDA stereotactic intracerebral injection of the right medial forebrain bundle. Contralateral rotation was induced by apomorphine intraperitoneal injection 3 weeks later. The validated PD rats were administered with L-DOPA and benserazide for 3 weeks to induce a rat model of dyskinesia. Involuntary movements (AIMs) were rated every 3 days during the treatment. (**B**) AIMs of LID rats at various times. At 30 min intervals, AIMs were rated for 60 s for each rat for a total of 180 min. For each AIM category, the scores were summed for each time point. Data are shown as mean ± SEM (*n* = 15). (**C**) The typical AIM of LID rats. The limb AIM was manifested by tremor of the distal extremity and grasp, and contraction of the shoulder muscles, with hemiballismus. The axial AIM is manifested by torsion of the head, neck, and upper torso, or even loss of balance. The locomotive aim was shown as turning in circles to the opposite side of the injury. (**D**,**E**) Western blot of TH in the striatum and substantia nigra (SN) and c-FOS, p-ERK, and ERK in the striatum of control, PD, LID, and (non-LID) NLID rats (*n* = 3–5). (**F**) Ratio of TH-positive cells in the brain of control, PD, LID, and NLID rats. TH-positive cells were significantly decreased in the striatum and substantia nigra of PD, LID, and NLID rats. (**G**) Immunostaining of TH in the brain of control, PD, LID, and NLID rats. * *p* < 0.05, ** *p* < 0.01, *** *p* < 0.001 vs. sham group; # *p* < 0.05, ## *p* < 0.01, ### *p* < 0.001 vs. PD group.

Similar to human LID, repeated levodopa therapy induced abnormal involuntary movements (AIMs) (including dystonia, hyperkinesia, and/or stereotypies) in the PD model rats [15]. Starting 3 days after the turning behavior test, PD rats received methyl L-DOPA (6 mg/kg, 5 mg/mL; D9628-5G, Sigma-Aldrich) and peripheral decarboxylase inhibitor, benserazide (B7283-1G, Sigma-Aldrich, 12 mg/kg, 10 mg/mL) using single daily intraperitoneal injections for 21 days. The 6-OHDA-lesioned control rats received saline using the same protocol (Figure 1A). AIMs were rated every 3 days after L-DOPA therapy six times in 180 min [16]. For each AIM category (exhibition of axial, limb, oral-lingual, and locomotor movements), a severity score of 0−4 was assigned and summed for each time point. Rats with average AIMs >12 were assigned to the LID group, whereas those with no apparent dyskinesia and average AIM scores ≤12 constituted the NLID group. After the final AIM rating, the rats were euthanized for analyses. The control rats were fed for the same time without treatment and were euthanized.

### 2.3. Immunofluorescence Analysis

Rat brains were fixed with 4% paraformaldehyde and embedded with paraffin. Tissue sections (thickness) containing substantia nigra and striatum were incubated overnight at 4 °C with anti-tyrosine hydroxylase (TH) antibody (ab112, 1:700; Abcam, Cambridge, MA, USA). Immunolabeled sections were washed and incubated with goat secondary antibodies conjugated with Alexa Fluor 488 (ab150129, Merck Biosciences, Nottingham, UK). Sections were mounted with medium containing diamidino-2-phenylindole (DAPI) (H1200-10, Vector Laboratories, Burlingame, CA, USA). The images were analyzed using Pannoramic Viewer software (3D HISTECH, Budapest, Hungary).

### 2.4. Western Blotting

Striatum proteins of the Sham, PD, NLID, and LID rats were extracted using a protein extraction kit (GPP1814; GenePool, Beijing, China). Rabbit polyclonal anti-TH (ab112; 1:200), rabbit polyclonal anti-c-FOS (ab7963; 1:500) (both from Abcam); rabbit monoclonal anti-ERK1/2 (#4695; 1:1000), and rabbit monoclonal anti-p-ERK1/2 (#4377; 1:500) (both from Cell Signaling Technology, Danvers, MA, USA) were used as the primary antibodies. Rabbit monoclonal anti-GAPDH antibody (ab181602; 1:3000; Abcam) was used for the loading control. Protein band density was quantified using the Quantity One software (version 4.6.2; Bio-Rad, Hercules, CA, USA).

### 2.5. CircRNAs Extraction and Sequencing

Total RNA was extracted from the right striatum of LID and NLID rats using an RNeasy mini kit (Qiagen, Hilden, Germany) according to the manufacturer’s instructions. Strand-specific libraries were prepared using the TruSeq Stranded Total RNA Sample Preparation kit (Illumina, San Diego, CA, USA). Qubit 2.0 fluorometry (Life Technologies, Carlsbad, CA, USA) was used to quantify the purified libraries. An Agilent 2100 bioanalyzer (Agilent Technologies, Santa Clara, CA, USA) was used to confirm the insert size and calculate the molar concentration. The library was diluted to 10 pM and then sequenced on the Illumina HiSeq X-ten system. Library construction and sequencing were performed by Shanghai Biotechnology Corp. (Shanghai, China).

### 2.6. Differential Expression Analysis of circRNAs

Clean reads were obtained by filtering-out rRNA reads, adapters, short fragments, and other low-quality reads from raw reads using Seqtk. Fragments per kilobase of transcript per million fragments mapped (FPKM) was used as an index to measure the expression levels of transcripts. Q30 was calculated to evaluate sequencing accuracy. Clean reads were compared to the reference genome Rnor 6.0 using BWA-MEM [17]. Circular RNA candidates were predicted by CIRI computational pipelines [18]. Perl scripts were used to classify the predicted circRNAs. Counts of reads mapping across an identified backsplice were normalized by read length and number of reads mapping.

Differentially expressed genes between the LID and NLID groups were identified using edgeR [19]. The significance threshold (*p*-value) was determined using false discovery rate (FDR). The fold change was calculated according to the spliced reads per billion mapping value. Differentially expressed genes were filtered by the criteria of *p* ≤ 0.05 and fold change ≥2. The parental gene was obtained according to the position information of circRNAs.

### 2.7. GO and KEGG Analyses

Gene Ontology (GO) annotation and Kyoto Encyclopedia of Genes and Genomes (KEGG) pathway analysis were performed on the parental genes of differentially expressed circRNAs. Analysis of GO terms enrichment was performed using clusterProfiler [20]. The *p*-value and FDR of each function were calculated by Fisher’s exact test and multiple comparison test to screen out the significant function represented by different genes. The selection criteria for significant GO were *p* < 0.05. The KEGG pathways were assessed using KOBAS software [21].

### 2.8. qRT-PCR

Total RNA was extracted using the Ultrapure RNA Kit (CWbio, Beijing, China) and the remaining genomic DNA was digested using the DNase Ⅰ Kit (CWbio). The RNA samples were reverse-transcribed into cDNA by using the HiFi-MMLV cDNA First Strand Synthesis Kit (CWbio) according to the manufacturer’s instructions. The qRT-PCR was performed with UltraSYBR Mixture (CWbio). The sequences of primers were listed in Table 1. Each sample was run in triplicate. GAPDH was used as a reference and the relative expression levels were calculated with the 2^−ΔΔCt^ method.

### 2.9. Competing Endogenous RNAs Network

The CeRNA (competing endogenous RNA) network was constructed based on the relationships between circRNAs, miRNAs, and mRNAs. The miRNA binding sites on the circRNAs and target genes of miRNAs were analyzed using miRanda and TargetScan systems [22]. The potential target mRNAs were predicted by TargetScan and miRDB (http://www.mirdb.org/ accessed on 1 May 2022) [23]. Cytoscape (version 3.8.2; www.cytoscape.org, accessed on 1 May 2022) was used to build the network.

### 2.10. Fluorescence In Situ Hybridization (FISH)

FISH was performed to detect the subcellular location of rno-Rsf1_0012. The brain tissue was incubated in the fixative for 12 h, then dehydrated by gradient alcohol, followed by paraffin treatment and embedding. The paraffin sections (thickness) were sliced and incubated in a 62 °C oven for 2 h. Xylene and ethanol were used for dewaxing and dehydration, respectively. The slices were boiled in the retrieval solution for 10−15 min and cooled to room temperature. The tissue was digested with proteinase K (20 μg/mL) at 37 °C for 20 min. Endogenous peroxidase was blocked with 3% methanol-H_2_O_2_. After prehybridization, a rno-Rsf1_0012 probe hybridize solution with a concentration of 1 μM was added to each section and the sections were incubated in a humidity chamber and hybridized overnight at 42 °C. Blocking solution (rabbit serum) was added to the section after removing the hybridization solution, then anti-DIG-HRP was added and incubated at 37 °C for 40 min. TSA chromogenic reagent was added to the labeled tissue and reacted in the dark for 5 min at room temperature. Cell nuclei were stained with DAPI for 8 min in the dark. The slides were observed with an Eclipse Ci (Nikon, Tokyo, Japan). The sequence of the rno-Rsf1_0012 probe was 5′-DIG-GCC TTT GGG TTT TAC TAG TTC TGG GTG ATT CG-DIG-3′.

### 2.11. Dual-Luciferase Reporter Assay

The interaction among circRNAs of interest and the predicted miRNA was confirmed using the dual-luciferase reporter assay. The mutant sequence fragments were assembled. To confirm the target binding, the wild-type sequence fragments of rno-Rsf1_0012 (wt) and the mutant sequence fragments of rno-Rsf1_0012 (mut) containing the estimated binding position were inserted into the pGL4.74 vector. The vectors and rno-miR-298-5p or mimics-NC were co-transfected into HEK293 cells. A dual-luciferase reporter gene assay kit (Beyotime Biotechnology, Shanghai, China) was used to detect the luciferase activity.

### 2.12. Statistical Analysis

Statistical analyses were performed using Prism 9 software (GraphPad, La Jolla, CA, USA). Data were compared by Student’s *t*-test (two groups) or by one-way ANOVA analysis of variance, followed by appropriate multiple comparisons tests (more than two groups). Data are expressed as the mean ± SEM.

## 3. Results

### 3.1. Validation of the LID Rat Models

SD rats were treated with apomorphine 3 weeks after surgery. Rats showing more than seven contralateral rotations/min were regarded as successful PD models. In this study, 43 SD rats were surgically treated, and 34 of them eventually became the PD model rats. The success of PD modeling was 79.1%. After chronic L-DOPA administration, 20 PD rats developed dyskinesia behavior and were assigned to the LID group (Figure 1C). Within 3 weeks, the AIM score of LID rats increased gradually (Figure 1B), while the control group treated with saline did not develop dyskinesia.

TH is a rate-limiting enzyme of catecholamine synthesis and a marker of dopaminergic neurons. TH activity, TH synthesis, and TH mRNA are decreased in the striatum of PD patients and animal models. TH immunofluorescence analysis of striatum and substantia nigra indicated that TH was significantly decreased in the ipsilateral side striatum and substantia nigra of PD, LID, and NLID rats, suggesting that dopaminergic neurons in the substantia nigra were lost and dopamine in the striatum was decreased after striatal 6-OHDA injection, while there was no significant change in the contralateral side (Figure 1F,G).

Western blot analysis of TH levels confirmed the results of TH immunohistochemistry; compared with the control group, the protein levels of TH of PD, LID, and NLID rats decreased significantly (Figure 1D,E). Immediate early genes (IEGs) can be activated transiently and rapidly in response to stimuli. IEGs coded proteins, including ΔFosB, FosB, and c-Fos are known as downstream signaling proteins of extracellular signal-regulated kinase (ERK) phosphorylation, which is hyperactivated in LID models and patients [24,25,26,27]. Therefore, we further assessed the expressions of c-Fos, phosphorylated (p-) ERK, and total ERK in the striatum of LID rats. As expected, c-Fos and p-ERK protein levels were reduced in PD rats compared with sham controls, while the expression levels of these two proteins in LID and NLID rats were significantly increased compared with PD rats, consistent with our previous report [28] (Figure 1D,E). Taken together, these results indicated that LID rat models were successfully established.

### 3.2. circRNAs Expression Profiles of LID and NLID Rats

**Figure 2:**
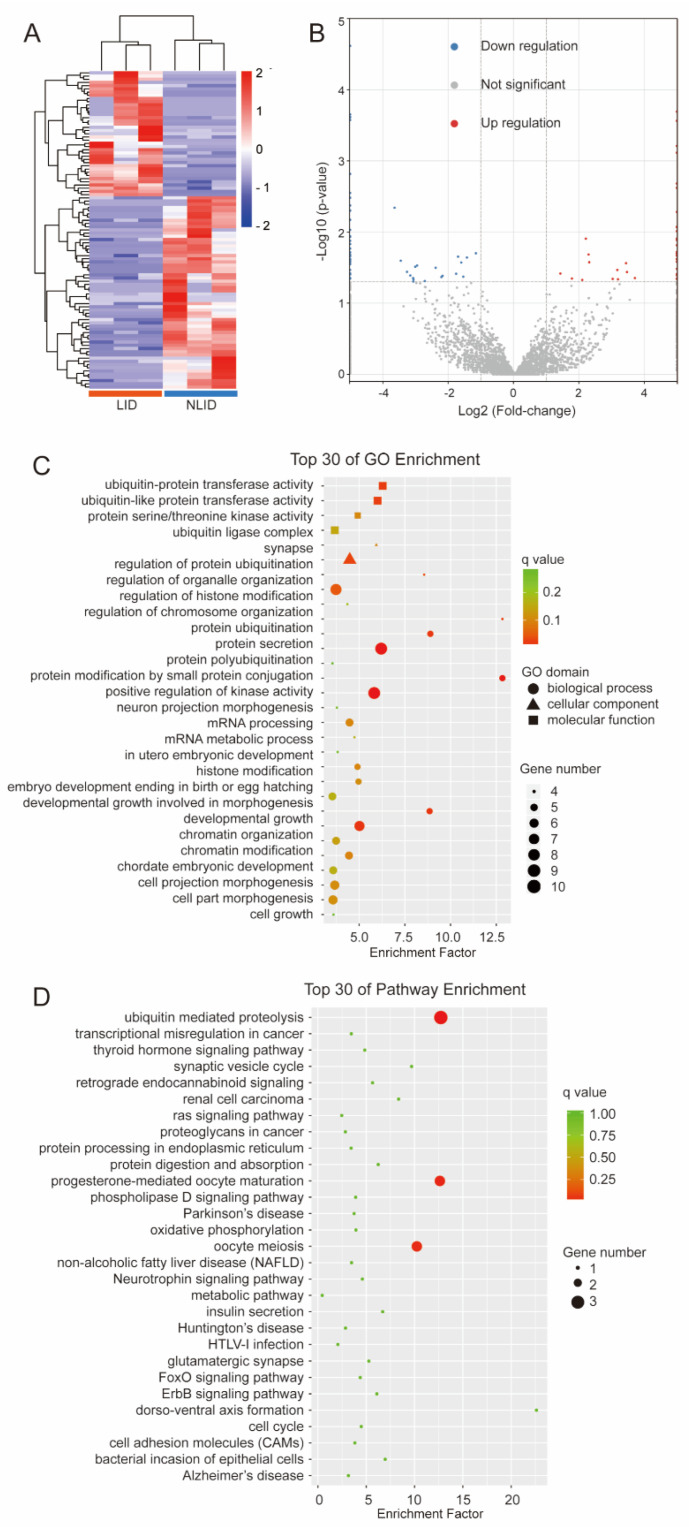
Expression of DEcircRNAs. (**A**) Hierarchical clustering of DEcircRNAs in LID (levodopa-induced dyskinesia) rats compared to non-LID (NLID) rats. The expression values are denoted using a color scale. The intensity increases from red (relatively higher expression) to blue (relatively lower expression). Different columns represent different samples (*n* = 6), and each row represents a single circRNA. (**B**) Volcano plot of circRNAs. A total of 39 up-regulated and 60 down-regulated circRNAs were screened out. Up-regulated and down-regulated circRNAs are denoted in red and blue, respectively. (**C**) Gene Ontology (GO) enrichment analysis. The abscissa is the rich factor, and the ordinate represents the GO terms. The size of the dot represents the number of genes annotated to the GO term. The color of the dot represents the q value. (**D**) Kyoto Encyclopedia of Genes and Genomes (KEGG) enrichment analysis. The size of the dot represents the number of genes annotated to the KEGG term. The color of the dot represents the q value.

Following 6-OHDA lesioning and L-DOPA administration, total RNA was isolated from striatum samples of three LID rats and three NLID rats for sequencing. The circRNAs and mRNAs that were differently expressed in LID and NLID rats were screened by high-throughput RNA sequencing to figure out the global circRNAs and mRNA landscape. In terms of the percentages of bases, they were evenly distributed in all samples. The percentages of clean reads in all samples were >93%, and the ratio of mapped reads of all samples was >92%. The reads derived from linear RNA and circRNA were unevenly located in all chromosomes, especially on ch1, ch2, and ch3 (Figure S1). The classification of predicted circRNAs indicated that most of them were ecircRNAs (Figure S2). According to the criterion of fold change ≥2 and *p* < 0.05, a total of 99 differentially expressed circRNAs (DEcircRNAs) were obtained between the LID and NLID groups, of which 39 were up-regulated and 60 were down-regulated (Table 2; Figure 2A,B).

### 3.3. Functional Annotation of the Host Genes of DEcircRNAs

GO enrichment analysis revealed that the host genes of DEcircRNAs mainly participated in protein ubiquitination (GO terms: protein ubiquitination, *p* = 2.38 × 10^−6^; protein polyubiquitination, *p* = 7.94 × 10^−6^; and regulation of protein ubiquitination, *p* = 2.72 × 10^−4^), neuronal morphology (GO terms: neuron projection morphogenesis, *p* = 1.01 × 10^−3^; cell projection morphogenesis, *p* = 1.69 × 10^−3^; and cell part morphogenesis, *p* = 1.99 × 10^−3^), and histone modification (GO terms: regulation of histone modification, *p* = 4.26 × 10^−5^; and histone modification, *p* = 1.29 × 10^−3^) (Figure 2C and Figure S3). KEGG annotation and enrichment revealed that the host genes of DEcircRNAs mainly participated in pathways of ubiquitin-mediated proteolysis (*p* = 3.99 × 10^−4^) (Figure 2D and Figure S4).

### 3.4. Rno-Rsf1_0012 Expression Validated by qRT-PCR

**Figure 3:**
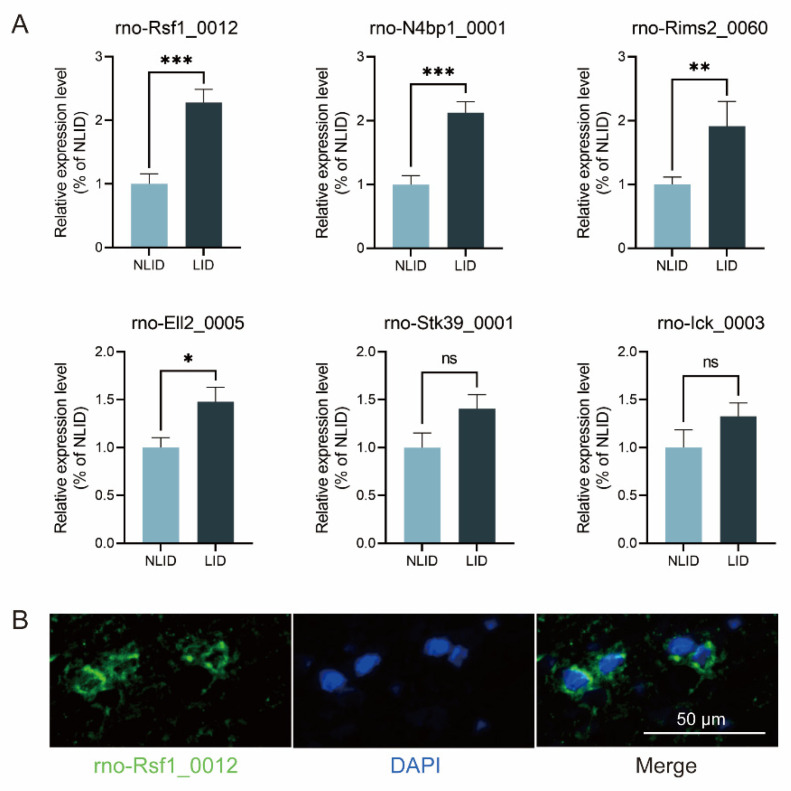
Expression profiles of rno-Rsf1_0012. (**A**) Validation of RNA sequencing results by qRT-PCR of six circRNAs. CircRNA rno-Rsf1_0012, rno-N4bp1_0001, rno-Rims2_0060, and rno-Ell2_0005 were significantly higher in the LID group (*n* = 6) than in the NLID group (*n* = 6). (**B**) Expression location of rno-Rsf1_0012. Immunofluorescence labeling of rno-Rsf1_0012 (green) and neuron markers (blue) in the striatum. * *p* < 0.05, ** *p* < 0.01, *** *p* < 0.001, ns non-significant.

Six most significant differential expressed up-regulated circRNAs (rno-Ick_0003, rno-N4bp1_0001, rno-Ell2_0005, rno-Rims2_0060, rno-Stk39_0001, and rno-Rsf1_0012) were selected for validation according to their functional annotations. Their expressions were analyzed by quantitative real-time (qRT-) PCR (Figure 3A). The PCR results showed that rno-Rsf1_0012, rno-N4bp1_0001, rno-Rims2_0060, and rno-Ell2_0005 were significantly higher in the LID group than in the NLID group, while there was no such difference in rno-Stk39_0001 and rno-Ick_0003. Among them, the difference of rno-Rsf1_0012 was the most significant, so subsequent studies focused on it. The mature sequence of rno-Rsf1_0012 was GTA AAA CCC AAA GGC AAA GTT CGA TGG ACT GGC TCT CGG ACA CGT GGC AGG TGG AAA TAC TCC AGC AAT GAT GAG AGC GAA GGG TCC GAG AGT GAC AAA TCC TCT GCC GCC TCG GAA GAG GAG GAA GGA AAG GAG AGT GAA GAA GCA GTC CTT CCA GAT GAC GAT GAA CCC TGC AAA AAG TGT GGC CTT CCG AAT CAC CCA GAA CTA. Fluorescence in situ hybridization (FISH) analysis showed that rno-Rsf1_0012 was mainly localized in the cytoplasm of neurons (Figure 3B).

### 3.5. Rno-Rsf1_0012 Regulates Expression of Target Genes via rno-miR-298-5p

**Figure 4:**
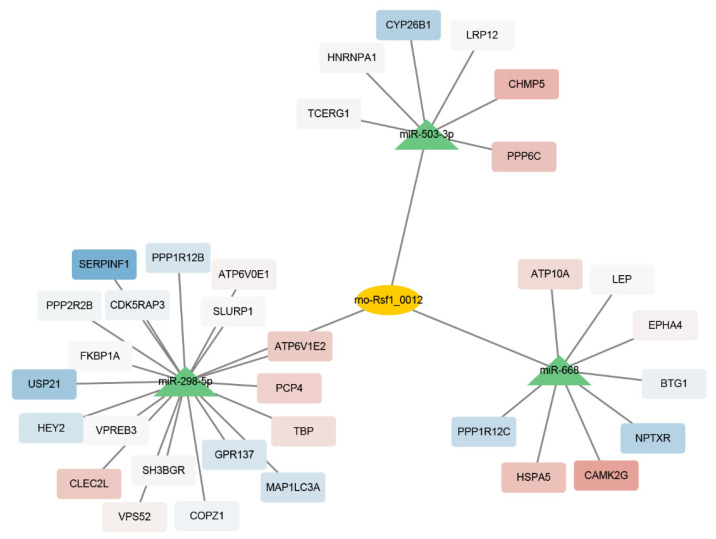
ceRNA network of rno-Rsf1_0012 with mRNAs. CircRNA rno-Rsf1_0012 and binding miRNAs are denoted by orange ellipse and green triangles, respectively. According to the trend of mRNA differential expression, the up-regulated and down-regulated mRNAs are denoted by red and blue rectangles, respectively.

**Figure 5:**
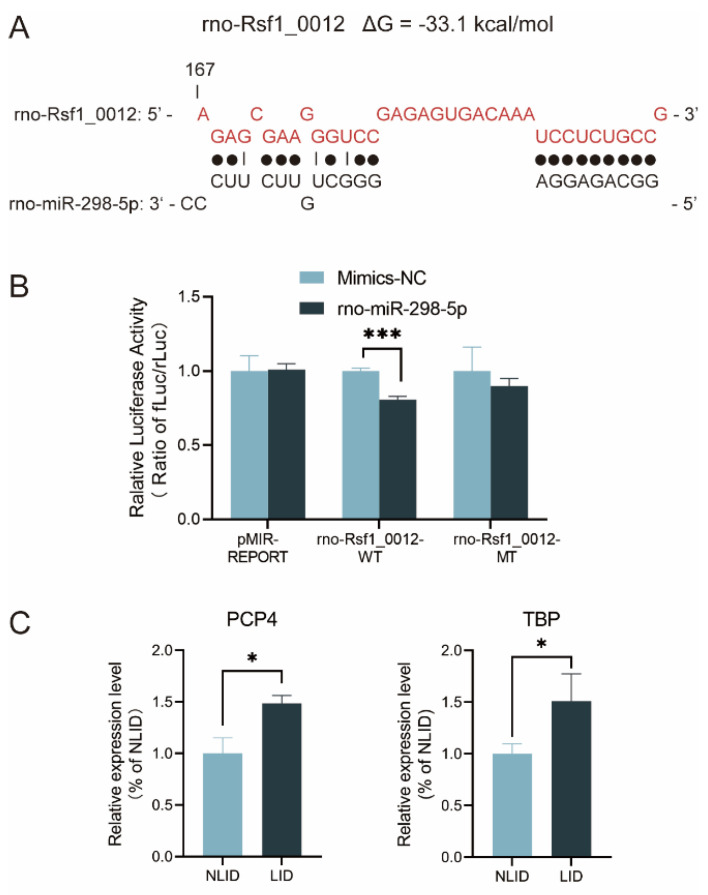
Dual-luciferase reporter assay and target genes expression. (**A**) Wild-type (WT) and mutated-type (MT) sequences of the putative binding sites between rno-Rsf1_0012 and rno-miR-298-5p. (**B**) Dual-luciferase reporter assays were performed to validate the association of rno-Rsf1_0012 and rno-miR-298-5p. Compared with the NC mimics, rno-miR-298-5p significantly reduced the luciferase activity of the WT reporter, while rno-miR-298-5p did not affect the luciferase activity of the MT reporter. (**C**) PCP4 and TBP in LID group were significantly increased in LID group (*n* = 6) compared with NLID group (*n* = 6). * *p* < 0.05, *** *p* < 0.001.

CeRNA mechanisms include the RNA transcript competitively binding miRNA, resulting in diluting the concentrations of free miRNAs in cells, reducing the inhibition of miRNA on coding RNA, and increasing the expressions of target genes [29]. The ceRNA network analysis showed that rno-Rsf1_0012 regulated multiple target genes by sponging miRNAs, including rno-miR-298-5p, rno-miR-503-3p, and rno-miR-668 (Figure 4). Interaction of rno-Rsf1_0012 and rno-miR-298-5p was confirmed using the dual-luciferase reporter assay. Wild-type (WT) and mutant (MT) dual-luciferase reporter vectors of rno-Rsf1_0012 incorporating miRNA binding sites were constructed and co-transfected with miRNA mimics or NC mimics into HEK 293 cells (Figure 5A). Compared with the NC mimics, rno-miR-298-5p significantly reduced the luciferase activity of the WT reporter, while rno-miR-298-5p did not affect the luciferase activity of the MT reporter (Figure 5B). The expression of two target genes (PCP4 and TBP) was confirmed using qRT-PCR. Compared with the NLID group, the expressions of PCP4 and TBP in the LID group were significantly increased (Figure 5C). These results indicated that rno-Rsf1_0012 may function as a molecular sponge of rno-miR-298-5p using its predicted binding sites.

## 4. Discussion

Here we established a LID rat model using the well-recognized intraperitoneal injection of L-DOPA and benserazide after unilateral 6-OHDA lesioning of the nigrostriatal pathway. Because a subset of SD rats is LID resistant [30,31], we divided rats in our study into the LID and NLID groups according to whether the model was successful. High-throughput sequencing was then used to screen the DEcircRNAs in the striatum. Using bioinformatics approaches, we further narrowed the scope to focus on a subset of circRNAs that may have played a key regulatory role and found rno-Rsf1_0012 may be related to the occurrence and development of LID. This possibility was further verified by PCR results. According to the mechanism of ceRNA, we speculated that rno-Rsf1_0012 might function as miRNA sponges to abrogate the inhibitory impact of rno-miR-298-5p on target genes. The dual-luciferase reporter genes experiment confirmed this idea.

Although no animal model can fully replicate human PD, the unilateral striatum of SD rats damaged by 6-OHDA is one of the most well-studied PD models. The substantia nigra dopaminergic neuron degeneration and loss, glial cell proliferation, substantia nigra and striatum TH activity, and dopamine decrease are similar to that of human PD. Apomorphine-induced rotation behavior can be used to quantify PD behavior and facilitate evaluation by researchers. However, it must be noted that this model belongs to an acute injury model and cannot simulate the characteristics of the chronic progressive course of PD in humans. Human LID can be divided into different subtypes with different clinical manifestations and mechanisms, including peak-dose dyskinesia, diphasic dyskinesia, and “off” dystonia [32]. The widely used LID rat model adopted in this study can simulate the peak-dose dyskinesia well [33,34]. A certain percentage of SD rats showed resistance to L-DOPA during the preparation of the LID rat model [35]. Therefore, studying the differences between LID susceptible and LID resistant SD rats may be important in clarifying the molecular mechanism of LID. Starting from this phenomenon, Manfredsson et al. characterized the key regulatory role of Nurr1 in LID [36]. In the present study, the incidence and severity of AIMs in rats increased with an increase in levodopa dose, which was consistent with the phenomenon reported in LID patients [37].

The details of LID pathogenic mechanisms are not completely understood, similar to PD mechanisms. According to the classic neural circuit model, contrary to the formation of PD, after long-term administration of levodopa, the direct pathway is hyperactive, and the indirect pathway is inhibited. However, this general model cannot explain all the clinical phenomena and experimental results of LID. LID is associated with molecular changes mediated by dopamine D1 receptors in the striatum, including phosphorylation of ERK, MSK1, and histone H3 at the level of the medium spiny neuron of the striatal nigral pathway [26]. Activation of D1/DARPP32 by levodopa induces the translocation of phosphorylated ERK in the nucleus and subsequent activation of MSK1, which plays a key role in regulating synaptic plasticity and transcriptional activity [38].

Functional analysis showed that many host genes of DEcircRNAs such as N4bp1, Ubr5, Klhl2, Rc3h2, Ankib1, Ccnc, Birc6, Trip12, Cdc23, and Kdm2a were involved in ubiquitination. Previous studies have shown that the process of neuronal death in PD is related to ubiquitination, but there is no evidence that ubiquitination is involved in the pathological process of LID [39].

Rno-Rsf1_0012 is located on chr1 and is mainly expressed in brain tissue. There are no reports on the expression difference and functional verification of rno-Rsf1_0012 in PD or LID. Rno-Rsf1_0012 was mainly localized in the cytoplasm of neurons, which indicated that it might act in a ceRNA manner.

CircSNCA can increase SNCA expression by down-regulating miR-7 and inducing apoptosis in PD [40]. a-Synuclein is prone to aggregate protein which forms toxic aggregates and is a major component of Lewy bodies–hallmarks of PD [41]. Kumar et al. reported that circzip-2 may sponge miR-60-3p in the C. elegans model of PD [42]. However, the role of ceRNA in LID has been rarely reported. The interaction between rno-Rsf1_0012 and rno-mir-298-5p in this study was confirmed by dual-luciferase reporter gene assays, but still needs to be verified by functional experiments in vivo.

The target genes of rno-miR-298-5p, including PCP4 and TBP, are associated with some movement abnormality disorders such as Huntington’s disease and spinocerebellar ataxia. PCP4 encodes a neuron-specific calmodulin-binding protein and may play a role in the pathophysiology of Huntington’s disease and Alzheimer’s disease [43]. It is highly and specifically expressed in Purkinje cells altered by spinocerebellar ataxia type 2 progression [44,45]. Polyglutamine expansion in the TBP can cause spinocerebellar ataxia type 17 [46,47], which prompted us to further investigate the relationship between these target genes and LID in future studies.

## 5. Conclusions

In conclusion, the present study reveals that a set of circRNAs are differentially expressed between LID and NLID rats. Among them, rno-Rsf1_0012 is increased in LID rats and can regulate the expression of target genes by binding rno-miR-298-5p. Rno-Rsf1_0012 may play a vital role in LID occurrence, but its specific mechanism needs to be verified by subsequent function studies.

## Figures and Tables

**Table 1:** Primer sequences used for qRT-PCR.

ID		Sequence of Primers
rno-Rsf1_0012	Forward	5′-GCCTTCCGAATCACCCAGAA-3′
	Reverse	5′-GAATCCATTGACCGCTCATCAG-3′
rno-Rims2_0060	Forward	5′-GGCTCACAAGACAGGATTCTATT-3′
	Reverse	5′-GCTTTCTGTCTGAAGGCATGT-3′
rno-N4bp1_0001	Forward	5′-GCCATTACGAGTACATCAAAGGG-3′
	Reverse	5′-AACACAGAGGTCAGCACAAGTA-3′
rno-Ick_0003	Forward	5′-AAGGACTGGCGTTCATTCACA-3′
	Reverse	5′-GATGGCAGCACCAGCACAA-3′
rno-Stk39_0001	Forward	5′-GCTCTTCTCTGCTGGCTTGG-3′
	Reverse	5′-GGCTTACCTTGGCTTTCTGGAA-3′
rno-Ell2_0005	Forward	5′-GGTGGGTGCTTGTTAAGTATATTAC-3′
	Reverse	5′-GCTGCTTGATCTTCTGATATTCTTG-3′
PCP4	Forward	5′-CTCACTGCCAGAGGAGGAATG-3′
	Reverse	5′-AATTCTTCTTGGACCTTCTTCTGC-3′
TBP	Forward	5′-CTTCAGTCCAATGATGCCTTACG-3′
	Reverse	5′-CTGCTGCTGCTGCTGTCTT-3′

**Table 2:** Top 10 up- and down-regulated DEcircRNAs obtained by sequencing.

ID	Type	Fold Change	Regulation
rno-Ick_0003	exon	13.13123	UP
rno-N4bp1_0001	exon	11.10113	UP
rno-Ell2_0005	exon	10.88539	UP
rno-Rims2_0060	exon	9.183134	UP
rno-Stk39_0001	exon	9.045527	UP
rno-Rsf1_0012	exon	8.201328	UP
rno-Chd2_0001	exon	4.98735	UP
rno-Trip12_0024	exon	4.913605	UP
rno-Arl8b_0001	exon	4.636859	UP
rno-Dmd_0004	exon	4.296785	UP
rno-Ralgps2_0004	exon	6.565114	DOWN
rno-Susd1_0002	exon	7.722112	DOWN
circRNA.15164	intergenic region	8.034623	DOWN
rno-Sergef_0005	exon	8.351556	DOWN
circRNA.4818	exon	8.367079	DOWN
rno-Kdm4c_0013	exon	8.40449	DOWN
rno-Pcsk5_0002	exon	8.931029	DOWN
rno-Prex2_0027	exon	9.574243	DOWN
rno-Rps6ka5_0004	exon	10.93379	DOWN
rno-Slc16a10_0001	exon	12.47348	DOWN

## Data Availability

The datasets generated and/or analyzed during the current study are not publicly available due we have unpublished studies from this data but are available from the corresponding author on reasonable request.

## Supplementary Materials

The following supporting information can be downloaded at: https://www.mdpi.com/article/10.3390/brainsci12091206/s1, Figure S1: Genome distribution map of four samples; Figure S2: The classification of predicted circRNAs; Figure S3: Go functional classification of the host genes of DEcircRNAs; Figure S4: KEGG classification of the host genes of DEcircRNAs.
