# Supplementary material for: Transcriptome Sequencing Reveal That Rno-Rsf1_0012 Participates in Levodopa-Induced Dyskinesia in Parkinson’s Disease Rats via Binding to Rno-mir-298-5p
Source: Brain Sci. 2022 Sep 7;12(9):1206. doi: 10.3390/brainsci12091206 (PMC9496896; doi:10.3390/brainsci12091206)
Supplement: Supplementary file 1 [file brainsci-12-01206-s001.zip › brainsci-1884186-supplementary.pdf]

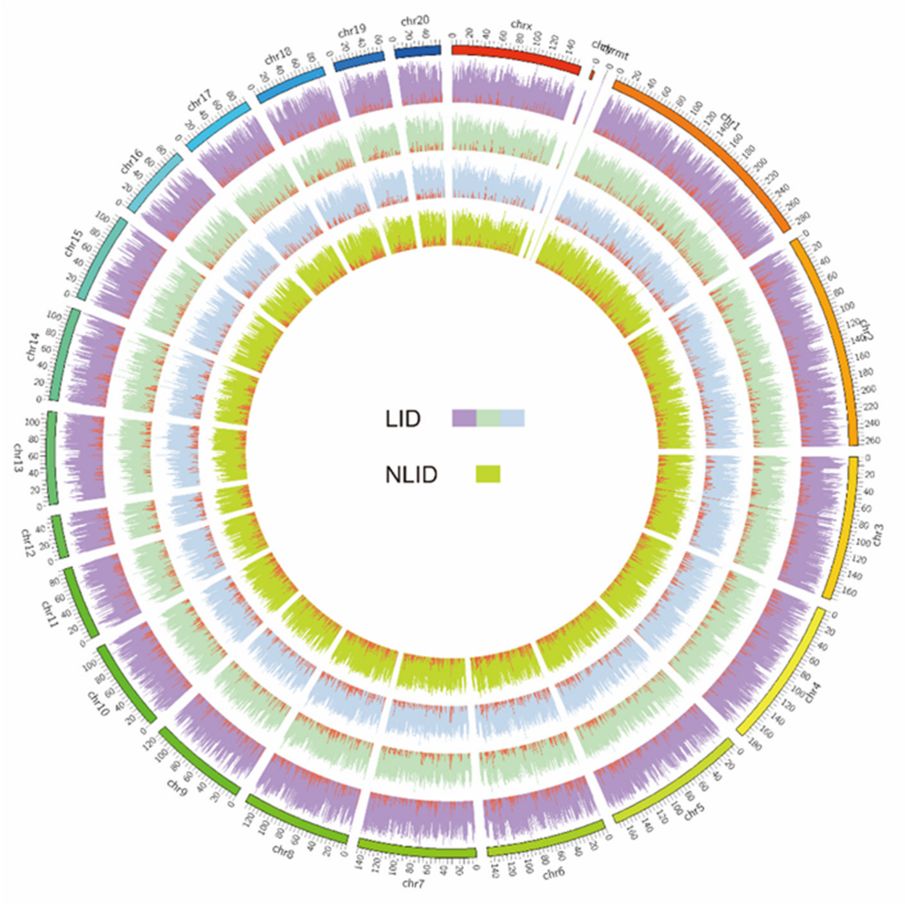

Figure S1. Genome distribution map of four samples.

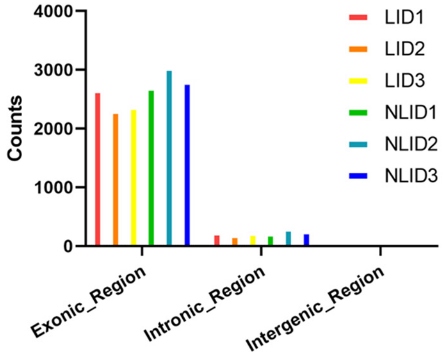

Figure S2. The classification of predicted circRNAs.

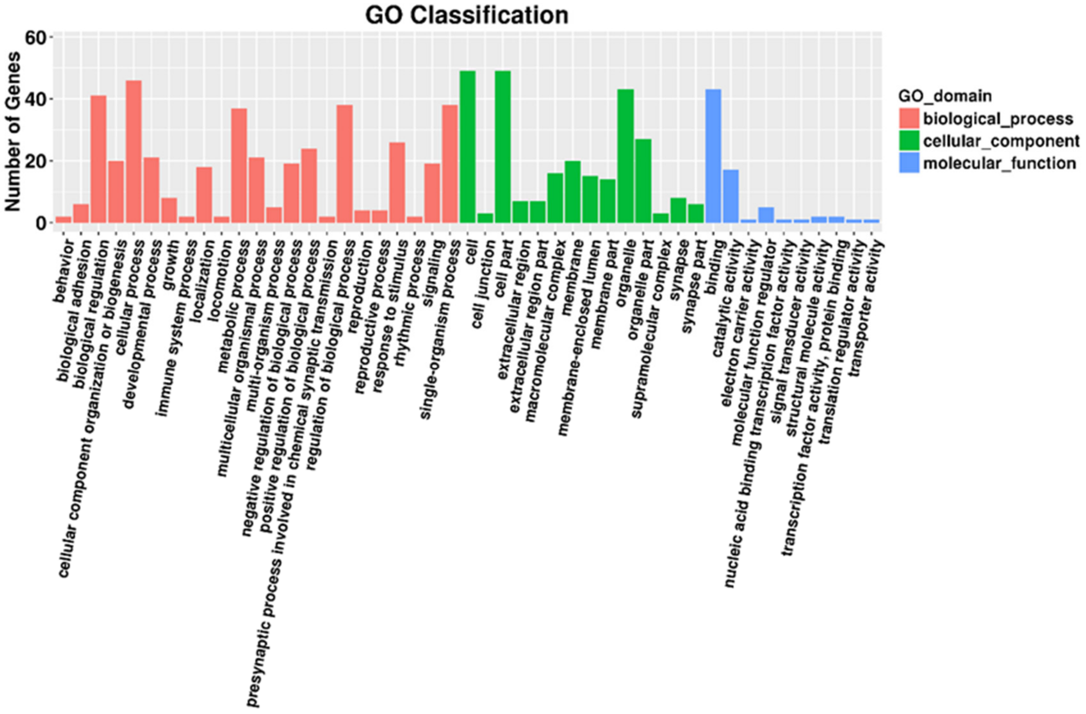

Figure S3. Go functional classification of the host genes of DEcircRNAs

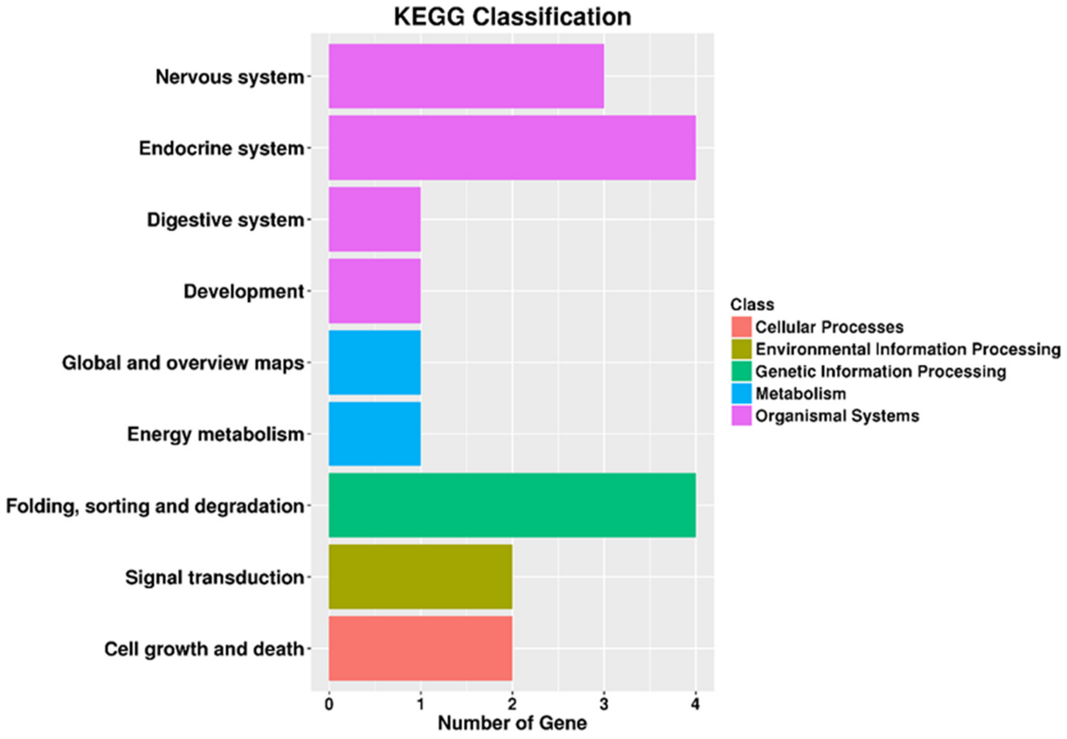

Figure S4. KEGG classification of the host genes of DEcircRNAs
